# Detection of *Streptococcus pneumoniae* from Different Types of Nasopharyngeal Swabs in Children

**DOI:** 10.1371/journal.pone.0068097

**Published:** 2013-06-26

**Authors:** Felix S. Dube, Mamadou Kaba, Elizabeth Whittaker, Heather J. Zar, Mark P. Nicol

**Affiliations:** 1 Division of Medical Microbiology, Faculty of Health Sciences, University of Cape Town, Cape Town, South Africa; 2 Institute for Infectious Diseases and Molecular Medicine, Faculty of Health Sciences, University of Cape Town, Cape Town, South Africa; 3 Academic Department of Paediatrics, Wright–Fleming Institute, Imperial College London, St Mary's Campus, London, United Kingdom; 4 Department of Paediatrics and Child Health, University of Cape Town, South Africa; 5 Red Cross War Memorial Children's Hospital, Cape Town, South Africa; 6 National Health Laboratory Service, Groote Schuur Hospital, Cape Town, South Africa; Centers for Disease Control & Prevention, United States of America

## Abstract

**Background:**

A better understanding of the epidemiology of nasopharyngeal carriage of *Streptococcus pneumoniae* is important to assess the impact of vaccination and the pathogenesis of pneumococcal disease. We compared the recovery of *S. pneumoniae* from nylon flocked, Dacron and rayon swabs.

**Methods:**

The recovery of *S. pneumoniae* from mocked specimens using flocked, Dacron and rayon swabs were compared by culture. The yield from paired nasopharyngeal (NP) samples obtained from healthy children sampled with flocked and Dacron swabs was also determined using culture and *lytA*-targeted real-time polymerase chain reaction (qPCR).

**Results:**

Using mock specimen, the percentage recovery of *S. pneumoniae* ATCC 49619 (serotype 19F) strain from the flocked swabs was 100%, while it was 41% from Dacron swabs and 7% from rayon swabs. Similar results were observed for *S. pneumoniae* serotypes 1 and 5. *S. pneumoniae* was cultured from 18 of 42 (43%) paired NP samples from the healthy children (median age 8 [interquartile range (IQR) 5–16] months). The median number of colony-forming units (CFU) recovered from flocked swabs was two-fold higher (8.8×10^4^ CFU/mL [IQR, 2.0×10^2^ – 4.0×10^5^ CFU/mL]) than Dacron swabs (3.7×10^4^ CFU/mL [IQR, 4.0×10^2^–3.2×10^5^ CFU/mL], *p* = 0.17). Using *lytA*-targeted qPCR from paired NP samples, the median copy number of *S. pneumoniae* detected from flocked swabs was significantly higher than from Dacron swabs (3.0×10^5^ genome copies/mL [IQR, 1.3×10^2^−1.8×10^6^] vs. 9.3×10^4^ genome copies/mL [IQR, 7.0×10^1^−1.1×10^6^]; *p* = 0.005).

**Conclusion:**

Flocked swabs released more *S. pneumoniae* compared to both Dacron and rayon swabs from mock specimens. Similarly, higher bacterial loads were detected by qPCR from flocked swabs compared with Dacron swabs from healthy children.

## Introduction

*Streptococcus pneumoniae* is the leading bacterial cause of childhood pneumonia worldwide [1], [2]. *S. pneumoniae* colonizes the nasopharynx of many healthy young children and only causes pneumonia in a small proportion of those colonized [3]–[6]. The timing of acquisition, intensity of colonization and interaction of *S. pneumoniae* with other respiratory pathogens are likely to be key determinants for progression to pneumonia [7]–[10]. Since nasopharyngeal (NP) colonization by *S. pneumoniae* is key to understanding the pathogenesis of pneumococcal disease and is increasingly used as an endpoint for pneumococcal vaccine studies, it is important to establish the optimal strategy for recovery *of S. pneumoniae* from NP specimens. Sampling of the NP may be achieved using an NP swab or aspirate. NP swabs are preferred as the procedure is simpler, quicker and better tolerated by children. The ideal swab would be highly absorbent, maintain the viability of microorganisms present, release most of the specimen material into culture broth or transport medium and not inhibit culture or nucleic acid amplification.

Traditionally used swabs, such as Dacron and rayon swabs are constructed by winding the respective fibres onto the tip of the swab shaft. This swab design may potentially trap a large proportion of clinical material in the fibre matrix, potentially reducing the recovery of microorganisms. Flocked swabs are constructed by electrostatically attaching flocked fibres onto their nylon-tipped surfaces which potentially results in improved specimen collection and more efficient release of specimen material [11]. In addition, flocked swabs have been used for the detection of respiratory viruses [12]–[16], *S.pneumoniae* [17]–[19], and other bacteria [20]–[22].

There are limited data comparing the recovery of *S. pneumoniae* using different types of swabs [22]. Only one study has compared the recovery of *S. pneumoniae* by culture and nucleic acid amplification from Dacron, calcium alginate and rayon swabs [23]. No published study has compared the more recently available flocked swabs (Copan Italia, Brescia, Italy) with other swab types for the recovery of *S. pneumoniae*. Therefore, the aim of the present study was to compare the yield from different types of swabs using culture and nucleic acid-based detection methods.

## Materials and Methods

### Pneumococcal recovery from mockspecimens using flocked, Dacron, and rayon NP swabs

American Type Culture Collection (ATCC 49619) strain of *S. pneumoniae* (serotype 19F) as well as clinical isolates of *S. pneumoniae* serotypes 1 and 5 (donation from the Respiratory and Meningeal Pathogens Research Unit, National Institute for Communicable Diseases, National Health Laboratory Service, Johannesburg, South Africa) were sub-cultured onto Columbia blood agar base with 2% agar, 5% horse blood and 4 µg/mL gentamicin (CAG) (Greenpoint Media Laboratory, National Health Laboratory Service, Cape Town, South Africa) and incubated at 37°C in 5% CO_2_ overnight. The resulting colonies were then inoculated into 10 mL Todd-Hewitt broth and incubated at 37°C in 5% CO_2_ for 3 hours to an optical density of 0.5 at 492 nm (which corresponds to an exponential phase of approximately 1.2×10^8^ CFU/mL, as determined previously [data not shown]).

Three different types of NP swabs were compared, the flocked swab (cat. no. 516C; Copan Italia, Brescia, Italy), Dacron swab (cat. no. MW151D; Medical Wire & Equipment, Corsham, United Kingdom) and rayon swab (cat. no. 160C; Copan Italia, Brescia, Italy). Log-phase cultures of each of the *S. pneumoniae* strains were serially diluted (10-fold) in phosphate buffered saline (PBS). For each of the *S. pneumoniae* strains, triplicate aliquots (20 µL) of the 10^−2^−10^−4^ dilutions were dispensed into 200 µL tubes. A single swab of each type was briefly placed into each of the triplicate aliquots of the above mentioned dilutions series until the inoculum was absorbed into the swab. This was performed for all swab types tested. The swabs were allowed to stand at room temperature for 10 minutes before transferring them into 1 mL of skim milk-tryptone-glucose-glycerol (STGG) transport medium. The composition of the STGG medium used in this study is based on the study published by O′Brien et al. [24]. After 30 minutes, the vials containing STGG and swabs were vortexed for 15 seconds, and 20 µL was inoculated onto CAG media and incubated at 37°C in 5% CO_2_ overnight. In order to simulate 100% release of *S. pneumoniae* strains (referred to as control) from an inoculated swab into the STGG media, 20 µL aliquots of the 10^−2^−10^−4^ dilutions were directly inoculated into 1 mL vials of STGG. Thereafter, 20 µL aliquots were directly inoculated onto CAG media. The plates were incubated at 37°C in 5% CO_2_ overnight and colony-forming units (CFU) determined. The percentage of *S. pneumoniae* recovery was calculated as the proportion of the mean CFU recovered from each swab type divided by the control (simulated 100% CFU recovered). The experiment was repeated on three different days.

### Pneumococcal recovery from healthy children using flocked and Dacron NP swabs

The in-vitro study was subsequently complemented by an evaluation of the recovery of *S. pneumoniae* from flocked and Dacron NP swabs from healthy children. Rayon swabs were not included in this comparison as previous in-vitro studies, including our own findings (below) had demonstrated that flocked and Dacron swabs were better than rayon swabs for *S. pneumoniae* release [12], [20], [25]–[27], and there are practical difficulties in performing a 3-way comparison. Consecutive healthy children undergoing elective surgery at Red Cross War Memorial Children's Hospital were enrolled. Swabs were obtained by a trained research nurse using a standardised procedure [28]. Paired NP swabs were obtained from each child from separate nostrils. A flocked swab was used to obtain a specimen from one nostril and a Dacron swab from the other nostril. The order of sampling was randomized. Written informed consent was obtained from a parent or legal guardian. The study was approved by the Human Research Ethics Committee of the Faculty of Health Sciences, University of Cape Town (HREC ref: 062/2011).

Following sampling, swabs were immediately placed into 1 mL of STGG, transported on ice to the laboratory and frozen at −80°C for later batch processing. After thawing, STGG samples were vortexed for 15 seconds to disperse organisms from the swab. A 10 µL aliquot was then inoculated onto CAG media and incubated at 37°C in 5% CO_2_ overnight.

### Total nucleic acid extraction from flocked and Dacron NP swabs from healthy children

The STGG aliquots were thawed to room temperature (22°C) and vortexed for 15 seconds. Thereafter, 300 µL of each sample was subjected to automated total nucleic acid extraction on the QIAsymphony SP instrument (Qiagen, Hilden, Germany) using the QIAsymphony® Virus/Bacteria mini kit (cat.no. 931036) according to the manufacturer's instructions. Total nucleic acid was eluted in 60 µL elution buffer and stored at −20°C until processing.

### Identification of *S. pneumoniae* using lytA-targeted real-time PCR from NP samples collected in healthy children

A quantitative real-time PCR (qPCR) assay for the detection of the *S. pneumoniae* autolysin-encoding gene (*lytA*) was performed to determine bacterial load recovered from either flocked or Dacron swabs collected from healthy children. Primers and probe used were those previously published by Carvalho et al. [29], and have been shown to be specific for *S. pneumoniae* detection [30]. The reaction mix contained 2.5 µL genomic DNA in a total reaction volume of 12.5 µL containing; 1×TaqMan® gene expression mastermix (Applied Biosystems, California, United States of America), 200 nM for each primer and probe. PCR amplification was performed on the Bio-Rad CFX96 Touch™ Real-Time PCR amplification system (Bio-Rad Laboratories, Hercules, CA, United States of America). The thermal cycling conditions consisted of an initial hot start of 50°C for 2 minutes, denaturation at 95°C for 10 minutes, followed by 40 amplification cycles of 95°C for 15 seconds, 60°C for 1 minute. The lowest limit of detection of the qPCR assay was 10 copies/mL as determined by inspection of a standard curve (10-fold serial dilution of *S. pneumoniae* ATCC 49619 strain genomic DNA). The quantification cycle (Cq) value at the detection limit point was 36. A positive (*S. pneumoniae* ATCC 49619) and non-template control (sterile water) were included in each run. Results below the lowest limit of detection were considered negative.

### Statistical analysis

Statistical analysis was performed using GraphPad Prism version 6.01 (GraphPad Software Inc, California, United States of America) and STATA software version 11.0 (Stata Corporation, Texas, United States of America). For normally distributed data, unpaired student t-test was used to compare the means of two groups. Wilcoxon rank-sum test was used to assess the median between two groups when the data was not normally distributed. Analysis of variance was performed to determine whether the mean CFU of *S. pneumoniae* recovered was different within triplicates of each swab type or across *S. pneumoniae* strains when the experiments were repeated on three different days. A *p* value less than 0.05 was considered as significant.

## Results

### Pneumococcal recovery from mock specimens by culture

Overall, there was a significant difference in the mean CFU of *S. pneumoniae* recovered across all the swab types (*p*<0.001) and across the three *S. pneumoniae* strains (*p* = 0.012) tested. There was no statistical differences in the mean CFU of *S. pneumoniae* recovered within triplicates of each swab type performed on the same day (*p* = 0.85) or when the experiments were repeated on three different days (*p* = 0.89), suggesting that the experiments were reproducible. As such, the *S. pneumoniae* CFU recovered from triplicates of each swab type and on three different days were pooled.

**Figure 1:**
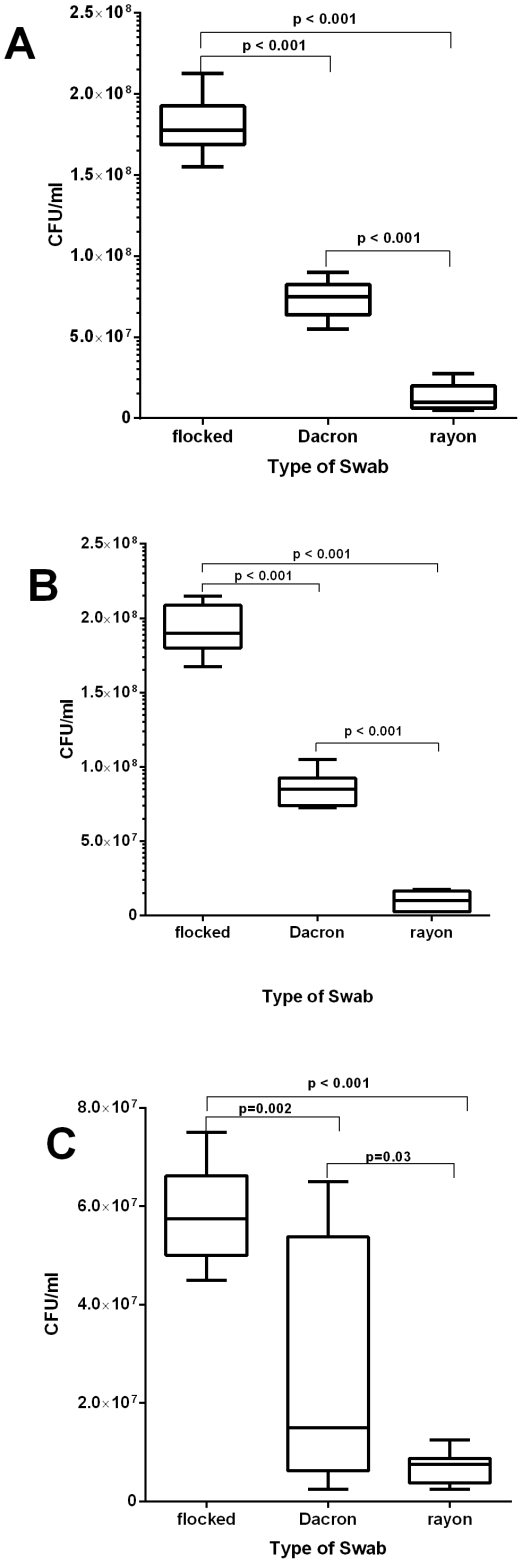
Recovery of *Streptococcus pneumoniae* from mock specimens using flocked, Dacron and rayon swabs. The data is presented as the pooled mean CFU recovered from 9 replicate of each swab type using *S.pneumoniae* serotype 19F (Fig 1A), serotype 1 (Fig 1B) or serotype 5 (Fig 1C). An unpaired student t-test was used for statistical comparisons.

The percentage recovery of *S. pneumoniae* ATCC 49619 (Serotype 19F) strain from flocked swabs was 100%, while it was 41% from Dacron swabs and 7% from rayon swabs (Table 1). The mean number CFU of *S. pneumoniae* recovered from the ATCC 49619 (serotype 19F) strain using flocked swabs was higher when compared with Dacron (18×10^7^ CFU/mL vs. 7.3×10^7^ CFU/mL, *p*<0.001) and rayon swabs (18×10^7^ CFU/mL vs. 1.3×10^7^ CFU/mL, *p*<0.001) (Fig. 1A). Dacron swabs released significantly more *S. pneumoniae* (ATCC 49619) than rayon swabs (7.3×10^7^ CFU/mL vs. 1.3×10^7^ CFU/mL, *p*<0.001). Similar results were also observed for *S. pneumoniae* serotypes 1 (Fig. 1B) and 5 (Fig. 1C).

**Table 1:** Recovery of *Streptococcus pneumoniae* from mock specimens using flocked, Dacron and rayon swabs.

*S. pneumoniae* strain (100% CFU recovery)^¥^	Swab type	Dilution	n^¶^	Mean CFU recovery/mL (SD)^‡^	Percentage of recovery ^†^
Serotype 19F^§^ (1.8×10^8^)	flocked	1/1000	9	18×10^7^ (5.7×10^6^)	100
	Dacron	1/1000	9	7.3×10^7^ (3.7×10^6^)	41
	rayon	1/1000	9	1.3×10^7^ (2.7×10^6^)	7
Serotype 1 (1.9×10^8^)	flocked	1/1000	9	19×10^7^ (5.4×10^6^)	100
	Dacron	1/1000	9	8.5×10^7^ (3.8×10^6^)	45
	rayon	1/1000	9	1.0×10^7^ (2.1×10^6^)	5
Serotype 5 (5.8×10^7^)	flocked	1/1000	9	5.8×10^7^ (3.2×10^6^)	100
	Dacron	1/1000	9	2.6×10^7^ (8.2×10^6^)	45
	rayon	1/1000	9	0.7×10^7^ (1.1×10^6^)	12

Anotype 19Frb type tested swab type from three the data wass) was determined by ica) as determined by the coefficient of variatSD, Standard deviation. ^¥^ Represents skim milk-tryptone-glucose-glycerol (STGG) media simulating a 100% release of *S. pneumoniae* from an inoculated swab into the STGG media and is used as a reference to calculate the percentage of recovery. ¶ Pooled replicates of each swab type from three independent experiments performed on three different days. **^‡^** ^aFU*arCFU/mL)^Mean of colony forming units (CFU) obtained from 9 different replicates of each swab type tested. **^†^** Percentage of recovery was calculated as the proportion of the mean CFU recovered from each swab type divided by the control (simulated 100% CFU recovery) from each respective *S. pneumoniae* serotypes. ^§^ *S. pneumoniae* serotype 19F tested in this study corresponds to the American Type Culture Collection (ATCC 49619) strain. [41]

### Pneumococcal recovery from NP samples collected from healthy children

#### Cultivation

Paired (flocked and Dacron) NP samples were collected from 42 healthy children (median age 8 [IQR 5–16] months) according to the WHO *S. pneumoniae* carriage protocol [28]. *S. pneumoniae* was cultured from 18 of the 42 (43%) NP samples. In 12 of 42 (29%) participants *S. pneumoniae* was recovered from both flocked and Dacron swabs, whilst in four (10%) and two (5%) participants *S. pneumoniae* was recovered only from flocked and Dacron swabs respectively. The children from whom *S. pneumoniae* was recovered were younger than those from whom it was not (8 months vs. 12 months, *p* = 0.032). Although the median number of CFU recovered from flocked swabs was approximately two-fold higher (8.8×10^4^ CFU/mL [IQR, 2.0×10^2^ – 4.0×10^5^ CFU/mL]) than that of Dacron swabs (3.7×10^4^ CFU/mL [IQR, 4.0×10^2^–3.2×10^5^ CFU/mL]), this difference was not significant, *p* = 0.17.

#### Quantitative real-time PCR

Using qPCR, *S. pneumoniae* was detected in 27 of the 42 (64%) NP samples overall, with similar detection rates from flocked and Dacron swabs (26 out of 42 [62%] vs. 24 out of 42 [57%]; *p* = 0.657). *S. pneumoniae* was detected in both flocked and Dacron swabs in 55% (23 out of 42) of the participants. In three participants, *S. pneumoniae* was detected from flocked swabs only and in one participant from Dacron swab only (*p*<10^−3^). Comparing the bacterial loads of *S. pneumoniae* detected by *lyt*A qPCR from paired NP samples, the median copy number of S. *pneumoniae* detected from flocked swabs (3.0×10^5^ genome copies/mL [IQR, 1.3×10^2^−1.8×10^6^]) was significantly higher than from Dacron swabs (9.3×10^4^ genome copies/mL [IQR, 7.0×10^1^−1.1×10^6^]; *p* = 0.005).

The comparison of *S. pneumoniae* detection by culture and qPCR from NP samples collected from healthy children is summarised in Table 2. The proportion of *S. pneumoniae* detected by either flocked or Dacron swabs by qPCR was higher when compared to bacterial culture (64% [27/42] vs. 43% [18/42]; *p* = 0.049).

**Table 2:** Comparison of *Streptococcus pneumoniae* detection by culture and real-time PCR from healthy children.

		Real-time PCR						
		flocked swab				Dacron swab		
		Positive	Negative	Total		Positive	Negative	Total
**Culture**	Positive	16 (38%)	0	16 (38%)		13 (31%)	1 (2%)	14 (33%)
	Negative	10 (24%)	16 (38%)	26 (62%)		11 (26%)	17 (41%)	28 (67%)
**Total**		26 (62%)	16 (38%)	42 (100%)		24 (57%)	18 (43%)	42 (100%)

PCR, Polymerase chain reaction targeting the autolysin gene (*lytA*) gene of *Streptococcus pneumoniae*.

## Discussion

A report from World Health Organisation (WHO) working group [28] and unpublished data from the US Centers for Disease Control and Prevention (CDC) [31] suggest the use of either Dacron or calcium alginate swabs collected in STGG transport medium for *S. pneumoniae* carriage studies. The present study demonstrated that flocked swabs were better compared to both Dacron and rayon swabs for recovery of *S. pneumoniae* from mock specimens. In addition, Dacron swabs recovered significantly more *S. pneumoniae* than rayon swabs. The findings from this study are in line with what has previously been shown for other bacteria [11], [21], [32]. For instance, Verhoeven et al. showed that flocked swabs were superior to rayon swabs for the recovery of *Staphylococcus aureus* using culture[20]. Similarly, flocked swabs have been shown to improve the recovery of epithelial cells and viruses compared to rayon swabs [12], [13], [16]. Our finding, that Dacron swabs were better than rayon swabs for the recovery of pneumococcus is in contrast with the finding of Rubin et al. (2008), following a similar protocol [23]. In our study, we used twice the volume of medium to inoculate swabs (20 µL vs. 10 µL) used by Rubin et al in their in-vitro assay [23].

Whilst we found no significant statistical difference between flocked and Dacron swabs for recovery of *S. pneumoniae* by bacterial culture from NP samples collected in children (*p* = 0.17), significantly greater bacterial load of *S. pneumoniae* was detected by qPCR from flocked swabs compared to Dacron swabs (*p* = 0.005). The importance of bacterial load has been recently demonstrated by Albrich et al. showing that the density of NP colonization by *S. pneumoniae* was higher in patients with pneumococcal community-acquired pneumonia compared to control patients using Dacron swabs for both culture and *lytA*-targeted qPCR [33]. Further, using the PCR-based methods, the severity of pneumonia caused by *S. pneumoniae* has been shown to be associated with an increased bacterial load in two independent studies using serum/blood samples [8], [34].

The present study did not assess the ability of each swab type tested for collecting other types of specimen, such as sampling of solid surfaces, vaginal and anal specimen collection. However, other studies have shown that flocked swabs are more efficient in recovery of bacteria (*e.g. Bacillus atrophaeus* spores, *S. aureus*, *Enterococcus hirae*) from solid surfaces [11], [32], [35], [36]. In addition, flocked swabs have been shown to collect similar or slightly more cells from anal specimen than Dacron swabs [37]. It has been shown that the DNA extraction methods may influence the DNA recovery from both cotton and flocked swabs used for the collection of DNA from saliva stains [38]. Therefore, in order to assess whether the observed high bacterial load in this study from flocked swabs is not inherent to the DNA extraction method, studies using different nucleic acid extraction methods are warranted.

In summary, our findings suggest that flocked swabs may offer an improved opportunity for recovery and detection of *S. pneumoniae* from NP swabs. Importantly, flocked swabs are increasingly used for NP sampling for detection of respiratory viruses by nucleic acid amplification [13], [15], [39], [40]. The use of a single swab type for both virological and bacterial studies would simplify specimen collection protocols [27].
